# Coexistence of anti-neurexin-3α-associated autoimmune encephalitis and systemic lupus erythematosus in an adult patient: a case report

**DOI:** 10.3389/fimmu.2025.1630292

**Published:** 2025-12-04

**Authors:** Huiting Zhang, Ning Kong, Zhimin Liao, Jiawu Fu, Jiangang Pan, Wangtao Zhong

**Affiliations:** 1 Department of Neurology, Affiliated Hospital of Guangdong Medical University, Zhanjiang, China; 2 First School of Clinical Medicine, Guangdong Medical University, Zhanjiang, China

**Keywords:** anti-neurexin-3α-associated autoimmune encephalitis, systemic autoimmune, abnormal behavior, consciousness disturbance, case report

## Abstract

Autoimmune encephalitis (AE) associated with anti-neurexin-3α antibodies, an uncommon variant of AE, exhibits particularly low frequency when coexisting with systemic immune dysregulation. The heterogeneous clinical manifestations of this neuroinflammatory disorder hinder timely diagnosis. We describe a 55-year-old male diagnosed with anti-neurexin-3α-associated AE, complicated by systemic lupus erythematosus (SLE) and antiphospholipid antibodies. The case presented with pyrexia persisting for 3 days, followed by acute-onset vertigo and encephalopathy evolving over 7 h. The clinical course was marked by visual obscuration, cephalalgia with associated emetic episodes, and concurrent neuropsychiatric manifestations, including psychomotor agitation and urinary incontinence. Anti-neurexin-3α antibody was detected in the cerebrospinal fluid. The patient tested positive for anti-neutrophil cytoplasmic antibody, anti-SS-A/RO60KD, anti-SS-A/RO52, anti-SnRNP, and lupus anticoagulant. Plasma complement levels (C3 and C4) were decreased, while elevated titers were observed for antinuclear antibodies, anti-double-stranded DNA, and anti-β2 glycoprotein I IgG antibodies. Immunomodulatory therapy with pulse methylprednisolone and intravenous immunoglobulin infusion elicited marked neurological recovery. This case underscores the imperative to investigate AE in the differential diagnosis of acute neuropsychiatric decompensation, and it is important to consider changes related to the aforementioned pathologies during physical examination and imaging evaluation.

## Introduction

Autoimmune encephalitis (AE) refers to rare neurological conditions involving brain inflammation due to immune system dysfunction. It has been demonstrated that the incidence of AE is about 1–2 per 100,000 people per year (1). AE is pathogenically linked to autoantibodies directed against neuronal surface antigens, synaptic adhesion molecules, voltage-gated ion channels, or neurotransmitter receptors. Antibody specificity dictates distinct neuroanatomical targets, with distinct epitope targeting driving pleomorphic clinical phenotypes through region-specific neurophysiological disruptions (2). In 2016, Gresa-Arribas et al. first included anti-neurexin-3α in the list of autoantibody targets for encephalitis (3). To our knowledge, only 12 cases of this encephalitis have been reported (3–10). Therefore, raising awareness of anti-neurexin-3α-associated AE remains highly valuable. The main clinical manifestations of anti-neurexin-3α-associated AE are epileptic seizures, memory and cognitive deficits, movement disorders, autonomic nerve dysfunction, and consciousness disturbance (3). Approximately half of the reported cases have presented systemic autoimmune abnormalities (11). In this study, we reported a novel case of anti-neurexin-3α AE with concurrent systemic lupus erythematosus (SLE) and abnormal antiphospholipid antibodies, thus broadening the clinical spectrum of diseases associated with neurexin-3α antibodies.

## Case presentation

On 1 January 2025, a 55-year-old male was urgently transferred to our tertiary care center following a 72 h history of pyrexia and acute-onset disequilibrium with progressive encephalopathic deterioration over 7 h. Three days prior to admission, the patient developed low-grade pyrexia (maximum 37.5 °C) secondary to a preceding upper respiratory tract infection. During initial management at a county hospital, transient focal motor seizures involving bilateral extremities were observed, characterized by absence of autonomic involvement and spontaneous resolution within 5 min. Recurrent stereotyped episodes (n = 3) were documented prior to escalation of care. The patient developed dizziness, blurred vision, headache, disorganized speech, irritability, unfamiliarity with family members, and nausea and vomiting, along with urinary incontinence, without limb weakness, dysphagia, or dysarthria. There were no complaints of ocular dryness, reduced lacrimation, or xerostomia. There was no history of arteriovenous thrombosis events. No rash was present on the face. The cranial nerve examination was unremarkable, with normal muscle strength and tone in all four limbs, absent bilateral pathological reflexes, and no signs of meningeal irritation. Non-contrast cranial computed tomography (CT) demonstrated multifocal calcific deposits within the left basal ganglia nuclei. Subsequent transfer to our tertiary neurosciences center was initiated for further diagnosis and therapy. The patient’s medical history was unremarkable, with negative documentation of allergic sensitization, prior surgical interventions, transfusion requirements, heritable neurological disorders, or chronic ethanol exposure.

**Figure 1:**
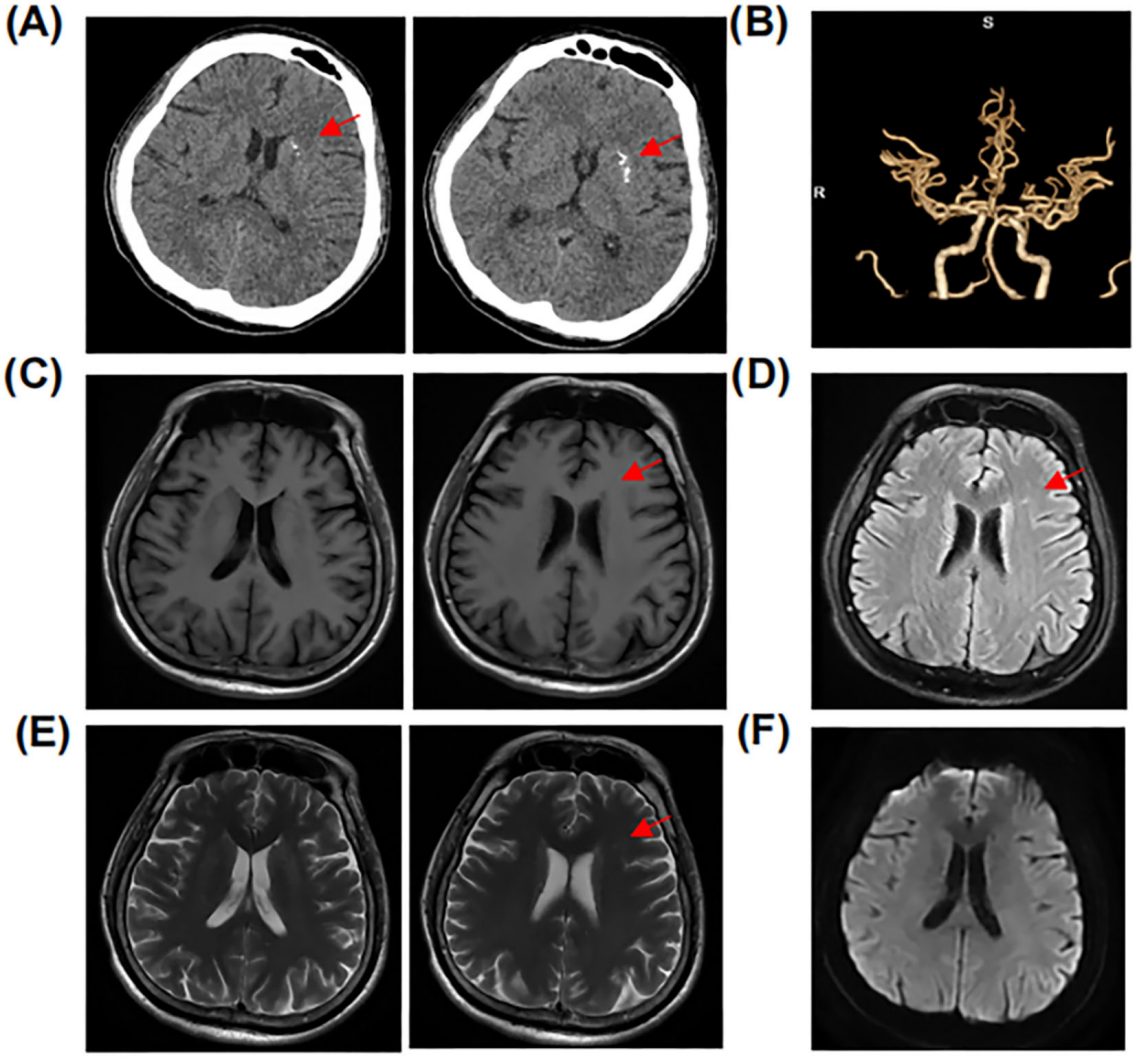
Cranial computed tomography (CT) and brain magnetic resonance imaging (MRI) scans of the patient. **(A)** Calcification in the left basal region. **(B)** Head computed tomography angiography (CTA) was unremarkable. **(C-F)** Brain magnetic resonance imaging (MRI) revealed a few punctate abnormal signals adjacent to the lateral ventricles and the sulci, fissures, and cisterns of the brain were widened.

**Figure 2:**
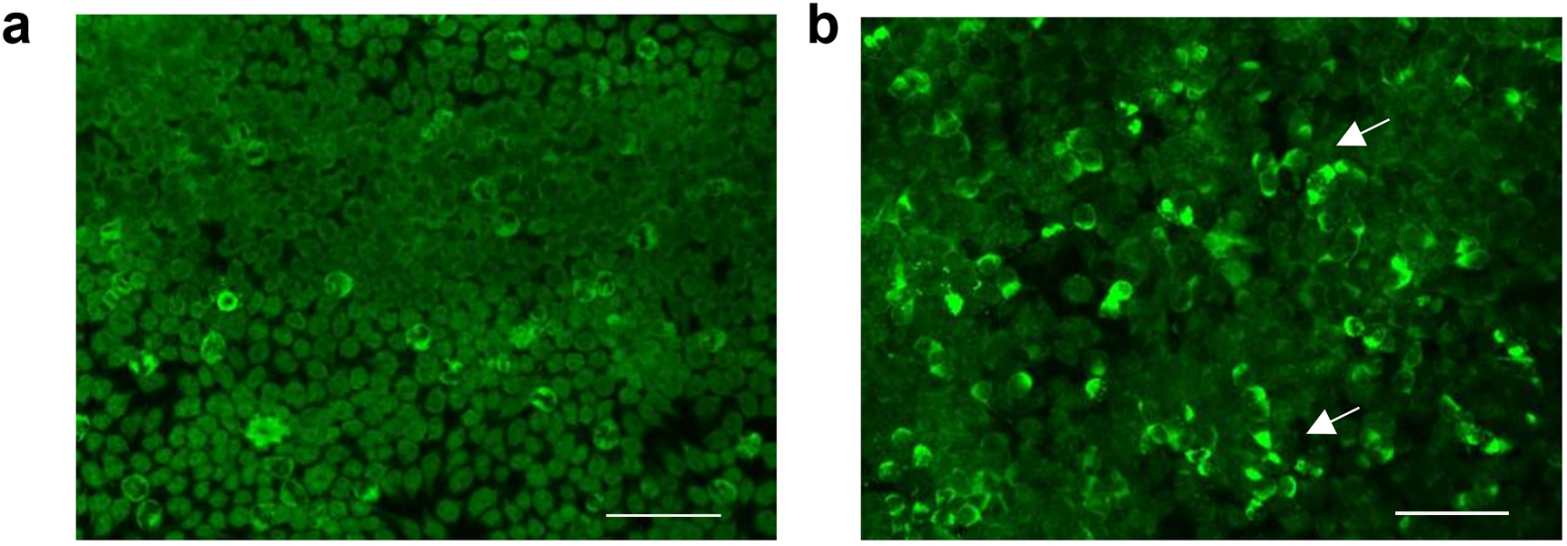
Anti-neurexin-3α antibody analyzed by cell-based assay (CBA) methods. **(A)** Immunofluorescence analysis revealed positivity in the experimental group (patient CSF co-cultured with HEK293T cells), as evidenced by green fluorescence, in contrast to control specimens **(B)**. Scale bar: 200μm.Scale bar: 200μm.

Upon initial neurological evaluation at our center, the patient demonstrated preserved arousal (Glasgow Coma Scale = 15) with profound cognitive–linguistic dissociation: fluent but dysarthric speech production, paraphasic errors, and impaired semantic processing manifesting as anosognosia for familial relationships. Neuropsychiatric assessment revealed a complex behavioral phenotype featuring (1): affective dysregulation with episodic vocal outbursts (2); multimodal hallucinations (auditory/visual) (3); sleep–wake cycle fragmentation; and (4) systematized paranoid ideation. Formal neurocognitive testing, including the Mini-Mental State Examination (MMSE) and Montreal Cognitive Assessment (MoCA), was precluded due to profound executive dysfunction. Laboratory tests were as follows: rheumatoid factor: 97 IU/ml (0–20 IU/ml), C-reactive protein (CRP): 26.01 mg/L (0–5 mg/L); erythrocyte sedimentation rate (ESR): 35 mm/h (0–26 mm/h); and procalcitonin: 0.051 ng/mL (<0.05 ng/mL). Complete blood count, serum ammonia, coagulation, and urinary tests showed no abnormalities. Serum cytomegalovirus, and herpes simplex virus type I antibody IgG antibodies were positive. Antinuclear antibodies (ANAs) were elevated at a titer of 1:640 (reference range: <1:80). Anti-neutrophil cytoplasmic antibody (ANCA), anti-SS-A/RO60KD, anti-SS-A/RO52, anti-SnRNP, and lupus anticoagulant were positive. Complement 3 (C3) was 0.524 g/L (reference range: 0.79–1.52 g/L), and complement 4 (C4) was 0.0832 g/L (reference range: 0.16–0.38 g/L). Anti-double-stranded DNA (anti-dsDNA) and anti-β2 glycoprotein I antibody IgG antibodies were elevated to 44.249 IU/mL and 25.7 CU, respectively. Serum tumor markers (alpha-fetoprotein, carcinoembryonic antigen, carbohydrate antigen-199, ferritin), anti-cardiolipin antibody, glycated hemoglobin, and thyroid function were normal. The patient met the 2012 Systemic Lupus International Collaborating Clinics (SLICC) criteria (12) and the 2019 European League Against Rheumatism/American College of Rheumatology classification criteria (13) for SLE, and the neuropsychiatric SLE (NPSLE) could not be ruled out. Brain CT showed a patchy high-density shadow is observed in the left basal region, with a CT value of approximately 146 Hounsfield units, suggesting a high likelihood of a calcification (**Figure 1A**). Computed tomography angiography (CTA) of the head showed no significant abnormalities (**Figure 1B**). Brain magnetic resonance imaging (MRI) revealed a few punctate abnormal signals adjacent to the lateral ventricles, with no enhancement. The sulci, fissures, and cisterns of the brain were widened (**Figure 1C–F**). Chest CT showed no abnormalities. Cerebrospinal fluid (CSF) appearance was clear and transparent. Lumbar puncture pressure was 200 mmH_2_O. Total cell count was 50×10^6^/L (reference range 0-8×10^6^/L), and white blood cell count was 30×10^6^/L (reference range 0-8×10^6^/L). Glucose and chloride levels were within the standard range. CSF bacterial culture, acid-fast bacilli staining, India ink staining, bacterial and viral cultures, immunoglobulins, and syphilis serology were unremarkable. The results of CSF metagenomic next-generation sequencing (mNGS) and serum paraneoplastic antibodies (Hu-IgG, Yo-IgG, Ri-IgG, CV2-IgG, Amphiphysin-IgG, Ma1-IgG, Ma2-IgG, SOX1-IgG, Tr/DNER-IgG, Zic4-IgG, PKCγ-IgG, Recoverin-IgG, Titin-IgG, GAD65-IgG) tested by dot enzyme-linked immunosorbent assays (Euroimmun, cat# D241024AP) were negative. CSF autoimmune encephalitis antibodies assessed using the tissue-based assay (TBA) and cell-based assay (CBA) methods (Hangzhou Sainting Medical Technology Co., Ltd., cat# 00116-240517) revealed positive anti-neurexin-3α IgG antibody (1:32; **Figure 2A, B**; **Supplementary Table S1**), while serum autoimmune encephalitis antibodies were negative.

The patient could not cooperate with the electroencephalographic monitoring. Definitive diagnoses of anti-neurexin-3α AE and SLE were established after rigorous exclusion of infectious, paraneoplastic, and metabolic etiologies (14, 15).

**Figure 3:**
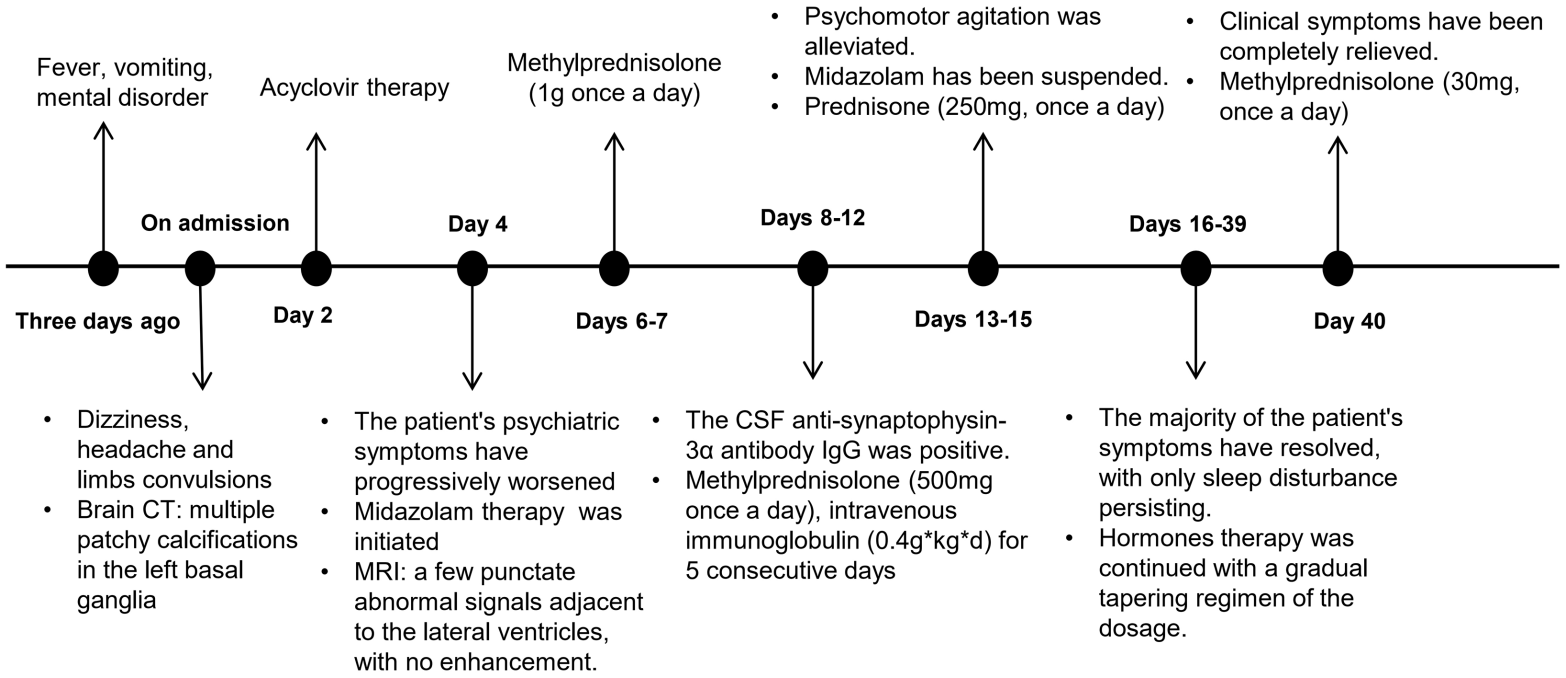
Diagnostic and therapeutic pathway of the patient.

Initial empirical antiviral therapy with acyclovir was discontinued upon serological confirmation of anti-neurexin-3α antibody-mediated encephalitis. The immunomodulatory protocol comprised pulse methylprednisolone therapy (1,000 mg/d iv×3d → 500 mg/d×2d) with concurrent intravenous immunoglobulin (0.4 g/kg/d×5d), followed by a corticosteroid tapering protocol: methylprednisolone 240 mg/d×3d transitioned to prednisone acetate (60 mg/d×14d) with subsequent biweekly 5 mg decrements. Psychomotor agitation was controlled using midazolam infusion initiated at 2 mg, with titration based on clinical response. Hemodynamic parameters and metabolic homeostasis were closely monitored and maintained within physiological ranges. The patient gradually improved, with complete cessation of perceptual disturbances, including auditory/visual hallucinations, and restoration of logical thought processes. The midazolam sedation protocol was successfully discontinued on day 7 post-immunotherapy initiation. On the 40th day of follow-up, the patient’s speech was clear, he was able to recognize his family members, and he showed no mental symptoms or insomnia. Maintenance immunosuppression with prednisone 40 mg/d continues per AE Consortium guidelines (15). The timeline of the patient’s condition is illustrated in **Figure 3**.

As shown in **Table 1**, we have summarized the characteristics of thirteen cases of anti-neurexin-3α-associated autoimmune encephalitis identified to date (including this case). The mean age of the patients was 44 years (range, 23–74), and 53.8% were female. More than half of the patients (9/13) presented with prodromal symptoms (headache, fever, nausea, or diarrhea) and experienced rapid progression (1–7 days, median 3) to memory decline, disability, seizure, and decreased level of consciousness. The majority of the patients (12/13) had underlying hematologic diseases or were found to have immune abnormalities, with the exception of one patient who had no other abnormal laboratory findings. Brain MRI revealed approximately 31% of cases with unremarkable structural neuroimaging, and 46% of the cases exhibited abnormalities in the hippocampus or temporal lobe. Among the thirteen patients, twelve patients exhibited neurexin-3α antibody positivity (CSF/serum) and concurrent CSF abnormalities, and one patient had no CSF data available (**Table 1**). All patients underwent first-line immunotherapy. Regarding to the therapeutic effect, there were three patients died (two from encephalitis/brain herniation secondary to encephalitis, and one died from sepsis), four patients had partial recovery, and four patients achieved full remission, and two patients experienced no marked improvement.

**Table 1:** Main clinical features of thirteen patients with anti-Neurexin-3α-associated autoimmune encephalitis.

Gender/Age	Prodromal symptoms	Major neurological symptoms	Other clinical features	MRI	CSF	Treatment	Prognosis
Female/23 (3)	Headache	Psychosis, lethargy, memory impairment, disorientation, and suspected central apnea.	Accompanied by leukopenia and brief thrombocytopenia.	Normal	1st: normal, 2nd: 20 WBC	Hormones	36-month follow-up: partial symptom recovery, residual memory deficits, anxiety, and nocturnal bi-level positive airway pressure.
Male/23 (3)	Headache, fever	Confusion, jealousy, restlessness, general convulsions, myoclonic jerks, and unconsciousness.	Accompanied by thrombocytopenia, leukopenia, antigen-antibody ratio of 1:60; post-mortem examination revealed: cerebral edema, cerebral hernia.	Normal	31WBC	Hormones	The patient died on the 17th day.
Female/50 (3)	Headache, fever, vomiting, diarrhea.	Abnormal behavior, seizures, decreased level of consciousness, mild facial movement disorders, and central respiratory insufficiency.	With a history of lupus and Raynaud’s disease	Abnormal signals on the medial temporal lobe FLAIR/T2 and DWI scans.	The IgG index increased.	Hormones + cyclophosphamide	4-month follow-up: partial symptom recovery, residual cognitive impairment, refractory epilepsy.
Female/44 (3)	Headache, fever	Insanity, restlessness, seizures, disconnected speech, and minor facial movement disorders.	Antigen-antibody 1:1280; Post-mortem examination suggests: mild subarachnoid hemorrhage, pulmonary findings suggest cytomegalovirus infection.	Normal	10 WBC	Hormones + immunoglobulin	Died on the 67th day, due to sepsis.
Female/44 (3)	Nausea, diarrhea	Confusion of consciousness, memory impairment, and seizures.	Accompanied by chronic joint pain and positive for thyroid protein antibodies.	Normal	24 WBC	Hormones	2-month follow-up: significant symptom improvement, with residual mild cognitive impairment (improving)
Male/57 (4)	Fever, weakness, dizziness, myalgia	Indifference, drowsiness, disruption of sleep-wake cycle, disorientation of consciousness, and changes in behavior.	The patient was diagnosed with malaria before the onset of symptoms.	Mild hyperintensity in the caudate-internal capsule cisterns.	159 Cell (91%NE)	Hormones	15 days later, symptoms were completely relieved.
Female/59 (5)	Confusion of consciousness	Decreased attention, impaired spatial orientation, and seizures.	Rheumatoid arthritis and Hashimoto’s thyroiditis.	Mild hyperintense in the left hippocampal.	Mild pleocytosis	Hormone + plasma exchange + rituximab	Full remission was achieved on Day 29.
Male/56 (6)	Fever, cough	Memory loss, seizures, myoclonic seizures, involuntary movements, dystonia, and autonomic nervous system dysfunction.	Normal	High signal intensity in bilateral temporal lobes, hippocampi, and insular cortices.	Normal CSF cell counts with elevated glucose level.	Hormones + immunoglobulin	Died 10 days later.
Male/58 (7)	Cognitive decline, depression	Seizures, memory loss, depressive mood, and slowed mental and motor function.	Positive for paraneoplastic Yo antibody and SOX1 antibody; family history of autoimmune diseases.	Normal	Elevated tau protein (70pg/ml).	Hormones	no marked improvement.
Female/21 (8)	Bradykinesia with hypophonia	Altered personality, social isolation, reduced verbal output, suicidal ideation, rigidity of extremities, bradykinesia, and gait instability.	Normal	Brain atrophy with symmetrical slight dilation of the ventricular system and widening of the sulci and ventricles.	14 WBC, elevated CSF protein.	Hormones + immunoglobulins + azathioprine	Hormone plus immunoglobulin treatment improved symptoms after 16 days, but relapsed after discontinuing treatment and improved again after re-treatment.
Female/74 (9)	None	Cognitive impairment, weakness in lower limbs	Brain biopsy: intracranial diffuse large B-cell lymphoma. 18FDG-PET/CT shows decreased metabolism in both the prefrontal and parietal lobes, and high activity in the medial-temporal lobe.	High signal intensity in right prefrontal and left parietal lobes and left medial-temporal lobe, with enhancement in right prefrontal lobe and left medial-temporal lobe.	Not mentioned	Hormones + immunoglobulins + plasma exchange	Not mentioned
Male/54 (10)	Episodic stinging in the left eye, weakness and numbness in right extremities	Generalized tonic-clonic seizures, agitation, auditory and visual hallucinations, cognitive decline, reduction in sleep duration.	Underwent left internal carotid stenting	Day 1: bilateral paraventricular demyelination Day 15: brain edema and meningitis	67 WBC, increased CSF Protein level (442.30mg/L)	Hormones + immunoglobulin + mortimycophenate + cyclophosphamide	Fully recovered at 7th month
Male/55 (this case)	Fever, vomiting, dizziness, headache.	Visual obscuration, cephalalgia with associated emetic episodes, concurrent neuropsychiatric manifestations.	Plasma levels of ANA, anti-dsDNA, rheumatoid factor were increased; Plasma ANCA, anti-SS-A/RO60KD, anti-SS-A/RO52, anti-SnRNP and lupus anticoagulant were positive.	A few punctate abnormal signals adjacent to the lateral ventricles, with no enhancement.	30 WBC	Hormones + immunoglobulin	Complete remission occurred on the 40th day.

## Discussion

Since the neurexin-3α antigenic epitope was identified in 2016, a total of 12 cases of anti-neurexin-3α AE have been documented worldwide (3–10). Among them, approximately half of the cases indicated the presence of systemic autoimmune abnormalities. The patient exhibited acute-onset encephalopathy with a prodromal febrile episode. Clinical manifestations included rapidly progressive cognitive dysfunction and neuropsychiatric disturbances, culminating in impaired consciousness and limb rigidity, suggestive of encephalitis. CSF analysis revealed IgG antibodies against neurexin-3α, fulfilling diagnostic criteria for AE (16, 17). The patient also met the diagnostic criteria for SLE, with concomitant Sjögren’s syndrome and antiphospholipid antibodies; a definitive diagnosis of anti-neurexin-3α AE associated with SLE was therefore established.

Neurexin-3α is a novel antibody target in AE, characterized as the predominant isoform within the synaptic vesicle glycoprotein superfamily, constituting 74% of total alternative splicing variants (18). Neurexins serve as critical presynaptic-postsynaptic interface mediators, modulating voltage-gated calcium channel activity and synaptic vesicle exocytosis via their evolutionarily conserved C-terminal domains (11, 19). Thereby, neurexin-3α demonstrates bidirectional neuromodulatory capacity through presynaptic vesicular release regulation and differential postsynaptic signaling integration, with region-specific functional heterogeneity observed across distinct brain areas (20). In the hippocampus, the extracellular sequence of presynaptic synaptic protein-3α affects memory and cognitive functions by mediating the trans-synaptic regulation of postsynaptic α-amino-3-hydroxy-5-methyl-4-isoxazole-propionic acid receptor (AMPAR). AMPAR functions as a pivotal molecular determinant in orchestrating enduring synaptic plasticity and mnemonic consolidation processes (21–23). Research has demonstrated that antibody-induced depletion of neuromodulin-3a disrupts synaptogenic maturation while preserving synaptic density, ultimately influencing the inhibitory and excitatory functions of synapses (24, 25). In this case, the patient exhibited memory decline and the difficulty in recognizing family members, which might be related to the interaction between neurexin-3a and AMPAR and synaptic function.

Anti-neurexin-3α-associated AE generally manifests with mental and behavioral disturbances, seizures, and cognitive decline. This distinct neuroimmune entity warrants critical diagnostic prioritization due to its frequent clinical overlap with alternative encephalitic pathologies, particularly anti-N-methyl-D-aspartate receptor (NMDAR) encephalitis and viral encephalitis. The definitive diagnosis of anti-neurexin-3α-associated AE necessitates rigorous integration of neurodiagnostic modalities, including clinical symptomatology, electroencephalographic anomalies, and brain MRI, with confirmatory serological and CSF autoantibody positivity. Notably, beyond established predictors such as advanced age and CSF protein elevation, dynamic prognostic determinants including refractory status epilepticus and neuroinflammatory-mediated cerebral edema may represent modifiable contributors to adverse clinical trajectories in neuroimmune encephalopathies (26–28). Previous studies have demonstrated that patients with positive systemic autoantibodies, particularly lupus antibodies, rheumatoid arthritis antibodies, and other related antibody markers, were prone to worsening conditions (3, 29). Notably, this patient manifested progressive worsening of clinical symptoms concurrent with systemic autoimmune abnormalities. Low complements (C3, C4), anti-SS-A/RO60KD, anti-SS-A/RO52, anti-SnRNP, and anti-dsDNA are highly indicative of SLE. Anti-SS-A/Ro52 and anti-SS-A/Ro60 are classic characteristics of Sjögren’s syndrome and are also are frequently present in SLE. Positive lupus anticoagulant and elevated anti-β2 glycoprotein I IgG are cardinal antibodies for antiphospholipid syndrome (APS). In the absence of clinical symptoms of Sjögren’s syndrome or thrombosis, we suppose that the positive anti-SS-A/Ro52, anti-SS-A/Ro60, and anti-β2 glycoprotein I IgG are considered concomitant serologic findings of SLE. These serological findings indicate that, rather than an isolated idiopathic AE, this case underscores the complex interplay between central nervous system (CNS)-specific and systemic autoimmunity. This permissive autoimmune environment may allow for the accidental generation of antibodies against neuronal surface antigens like neurexin-3α, a protein critical for synaptic adhesion and signaling. Secondly, systemic inflammation, a hallmark of active SLE, can disrupt the integrity of the blood–brain barrier (BBB). This breach allows the passage of pre-existing systemic autoantibodies and autoreactive immune cells into the CNS parenchyma. Anti-neurexin-3α antibodies can directly bind to their target on the neuronal surface, leading to internalization of the receptor and reversible synaptic dysfunction, which manifests as the clinical syndrome of encephalitis (seizures, psychosis, cognitive deficits). Thirdly, the initial inflammatory response driven by cell death and the release of nuclear antigens (e.g., DNA) can lead to ‘epitope spreading. Lastly, there is a fundamental loss of immune tolerance, leading to hyperreactivity of B and T cells against a wide array of self-antigens, both nuclear and cytoplasmic. As B cells, T cells, and the complement system are greatly involved in the pathological mechanisms of autoimmune diseases (30, 31). B-cell responses could be initiated against neuronal targets like neurexin-3α in a susceptible individual. We suppose that B cells, T cells, and the complement system might play a crucial role in the immune regulation targeting synapsin-3α. In summary, the finding of anti-neurexin-3α antibodies in a patient with this serological profile is best interpreted as a severe neurological manifestation of an underlying systemic autoimmune diathesis, most likely SLE with overlapping features of Sjögren’s syndrome and APS. Therefore, management must include acutely treating the encephalitis with immunotherapies (e.g., steroids, immunoglobulin) and concurrently managing the systemic disease with appropriate immunosuppressive regimens to achieve long-term remission and prevent further complications. Future research is awaited to explore the immunological mechanism of encephalitis, providing a theoretical basis for investigating additional monoclonal antibody therapies. First-line treatment for autoimmune encephalitis commonly includes intravenous immunoglobulin (IVIG), plasma exchange (PLEX), and corticosteroids. Monoclonal antibodies are part of the second line of treatment, so far. It is reported that approximately 60% patients with AE achieved a favorable prognosis following the aforementioned treatments (7). In this study, the patient achieved complete clinical remission following treatment with corticosteroids and human immunoglobulin.

In conclusion, anti-neurexin-3α AE can be associated with systemic autoimmune abnormalities, with concurrent autoimmune seropositivity portending adverse prognostic trajectories. The clinical manifestations of anti-neurexin-3α AE may be diverse, including mental and behavioral abnormalities, epileptic seizures, consciousness attenuation, and extrapyramidal dysfunction. Furthermore, the cranial MRI of anti-neurexin-3α AE may be negative, contributing to the difficulty in diagnosing this disease. Consequently, in addition to medical history, clinical manifestations, and MRI, the diagnosis of AE should focus on comprehensive screening of the immune antibody spectrum (e.g., neuronal surface antibodies, paraneoplastic antibodies) and and detection of systemic immune indicators. Relevant immunotherapy should be provided promptly, and second-line immunosuppressive treatment should be initiated when necessary.

## Data Availability

The datasets presented in this article are not readily available because of ethical and privacy restrictions. Requests to access the datasets should be directed to the corresponding authors.
