# Supplementary material for: Coexistence of anti-neurexin-3α-associated autoimmune encephalitis and systemic lupus erythematosus in an adult patient: a case report
Source: Front Immunol. 2025 Dec 4;16:1630292. doi: 10.3389/fimmu.2025.1630292 (PMC12711809; doi:10.3389/fimmu.2025.1630292)
Supplement: Supplementary file 1 [file Table1.docx]

**Table S1 The results of autoimmune encephalitis antibodies in CSF**

| Testing items | Result | Method |
| --- | --- | --- |
| NMDAR-IgG | （-） | CBA |
| AMPAR1-IgG | （-） | CBA |
| AMPAR2-IgG | （-） | CBA |
| LGI1-IgG | （-） | CBA |
| CASPR1-IgG | （-） | CBA |
| GABABR-IgG | （-） | CBA |
| DPPX-IgG | （-） | CBA |
| IGLON5-IgG | （-） | CBA |
| GlyRa1-IgG | （-） | CBA |
| GABAARa1-IgG | （-） | CBA |
| GABAARb3-IgG | （-） | CBA |
| mGluR5-IgG | （-） | CBA |
| D2R-IgG | （-） | CBA |
| **Neurexin3a-IgG** | **（+）** | **CBA** |
| GAD65-IgG | （-） | CBA |
| GABAARr2-IgG | （-） | CBA |
| Cerebellum | （-） | TBA |
| Hippocampus | （-） | TBA |
| cerebral cortex | （-） | TBA |
